# Influence of Mo addition on the structural and electrochemical performance of Ni-rich cathode material for lithium-ion batteries

**DOI:** 10.1038/s41598-020-64546-8

**Published:** 2020-05-22

**Authors:** Tahir Sattar, Seung-Hwan Lee, Bong-Soo Jin, Hyun-Soo Kim

**Affiliations:** 1 Next Generation Battery Research Center, Korea Electrotechnology Research Institute (KERI), Changwon-si, Gyeongsangnam-do, 51543, Republic of Korea; 2 Electro-Functionality Materials Engineering, University of Science and Technology (UST), 217 Gajeong-ro, Yuseong-gu, Daejeon 34113, Republic of Korea; 3 Faculty of Materials and Chemical Engineering, Ghulam Ishaq Khan Institute of Engineering Sciences and Technology, Topi, Khyber Pakhtunkhwa, Pakistan; 4 Department of Advanced Materials Engineering, Daejeon University, Daejeon, 34520, Republic of Korea

**Keywords:** Energy, Batteries

## Abstract

Molybdenum modified LiNi_0.84_Co_0.11_Mn_0.05_O_2_ cathode with different doping concentrations (0–5 wt.%) is successfully prepared and its electrochemical performances are investigated. It is demonstrated that molybdenum in LiNi_0.84_Co_0.11_Mn_0.05_O_2_ has a positive effect on structural stability and extraordinary electrochemical performances, including improved long-term cycling and high-rate capability. Among all samples, the 1 wt. % molybdenum LiNi_0.84_Co_0.11_Mn_0.05_O_2_ delivers superior initial discharge capacity of 205 mAh g^−1^ (0.1 C), cycling stability of 89.5% (0.5 C) and rate capability of 165 mAh g^−1^ (2 C) compared to those of others. Therefore, we can conclude that the 1 wt. % molybdenum is an effective strategy for Ni-rich LiNi_0.84_Co_0.11_Mn_0.05_O_2_ cathode used in lithium ion batteries.

## Introduction

Recently, the most widely used energy storage device is Lithium ion battery (LIB) due to its high energy density being able to fulfill the continuous demand for reducing the environmental impact and cost of electric vehicles and portable electronic devices^1–4^. For the cathode that determines the battery’s performance, a new family of cathode material; LiNi_x_Co_y_Mn_z_O_2_ (NCM) has attracted a lot of attention for the commercial applications^5,6^. NCM cathode possesses a complex cation arrangement which can be optimized to enhance cycle life, power density and thermal stability compared to traditional cathodes. Among the family of NCM cathodes, recent research efforts have been focused on Ni-rich (Ni≥80%) NCM cathodes because the increased Ni content brings low costs, high discharge capacity and energy density^7,8^. However, the low content of Co and Mn in Ni-rich NCM has a negative impact due to the thermally instability and cation mixing. It is reported that the delithiated Ni-rich cathode would undergo a phase transformation from layered to rock-salt during cycling which is closely related to the cation mixing^9–11^.

Cation mixing is caused by the auto-reduction of Ni^3+^ to Ni^2+^ which causes the collapse in the local structure due to which the Ni^2+^ migrates from transition metal slab to the lithium slab^12,13^. Also, Ni^3+/4+^ t_2g_ band overlaps with oxygen 2p band, that’s why high delithiation might result in the removal of electron from oxygen 2p band which causes the oxidation of O^2-^ and eventually loss of oxygen from the lattice^14,15^. A lot of techniques has been employed in order to suppress the cation mixing and improves the thermal stability and capacity. Coatings and doping are used to enhance the structural stability and electrochemical performance. Coatings of TiO_2_^16^, MgO^17^, SiO_2_^18^, ZrO_2_^19^ and Al_2_O_3_^20^ can minimize the contact area between the electrode and electrolyte and also suppress the oxygen release. However, these coatings have a drawback because they are electrically and electrochemically inactive^15^. Doping of Mg^21–23^, Na^13,24^, Al^25^, Ti^26^, Zn^27^ and Mo^28,29^ has not only stabilizes the structure but also improves the rate performance and cycling. Xue *et al*.^28^ has reported that Mo-doping in NCM-622 can reduce the Li/Ni cation mixing and Li-slab spacing is enlarged due to Mo migration which is beneficial for Li-ion diffusion. The doped sample suppresses the particle pulverization and reduces the charge transfer resistance which results in improved cyclic performance. Moreover, Konishi *et al*.^30^ has reported that Mo addition in NCM-811 ameliorates the thermal stability by suppressing the change in crystal structure.

To the best of our knowledge, very few studies has been done on the Mo-doping in Ni-rich (Ni≥80%) cathode material^28,29^. To clarify the effect of Mo-doping in LiNi_0.84_Co_0.11_Mn_0.05_O_2_ NCM cathode material (NCM84), we have done a detailed investigation on the structural and electrochemical properties. In the work, Mo-doping has been done in LiNi_0.84_Co_0.11_Mn_0.05_Mo_x_O_2_ (x = 0, 1, 3 and 5 wt.%) cathode materials. Mo-modified NCM cathode delivers the superior electrochemical performances. The 1% Mo-modified NCM84, exhibits excellent reliability and high performance capability.

## Experimental

**Figure 1:**
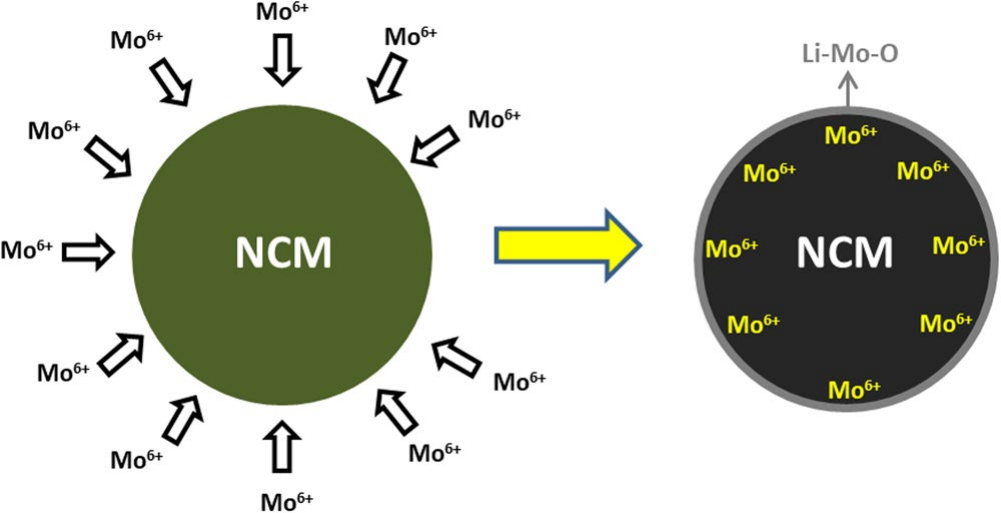
Schematic illustration of the synthesis process of Mo-modified NCM84.

The Ni_0.84_Co_0.11_Mn_0.05_(OH)_2_ precursor was supplied by Cosmo Materials Co., Republic of Korea. Precursor and LiOH were mixed in 1:1.05 ratio in acoustic mixer and in agate mortar and then pre-heated at 500 °C for 5 h, then calcined at 830 °C for 15 h while the heating and cooling rates were 5 °C/min. The calcination process was carried out in a tube furnace under air atmosphere. In addition, Mo-modified samples (Mo = 1.0, 3.0 and 5.0 wt. %) were synthesized by similar method. Stoichiometric amounts of precursor Ni_0.84_Co_0.11_Mn_0.05_(OH)_2_, LiOH and MoO_3_ were mixed and calcined at same heating cycle. The Mo-modified sample was prepared as illustrated in the Fig. 1. The un-modified sample was named as Mo-0 while the Mo-modified samples were Mo-1, Mo-3 and Mo-5 for the 1.0, 3.0 and 5.0 wt. %, respectively.

The samples morphology was analyzed by using field emission scanning electron microscope (FE-SEM, Hitachi S-4800). Structure and crystalline phase of the samples were characterized by X-ray powder diffraction (XRD, Philips, X-pert PRO MPD) with Cu Kα (λ = 0.15406 nm). The valence states of Ni, Co, Mn and Mg were determined by using X-ray photoelectron spectroscopy (XPS, VG MultiLab 2000) with Al Kα radiations.

The electrochemical test of assembled samples was measured with CR-2032 type coin cells. The cathode, consisting of active material (96%), carbon black (2%) as conductive material and polyvinylidene fluoride (PVDF, 2%) as binder were mixed in N-methyl pyrrolidinone (NMP) solvent to obtain homogeneous slurry. The slurry was coated on Aluminum foil and vacuum dried at 80 °C for 10 h. The loading rate was maintained at ∼15 mg/cm^2^ for all samples to meet the industry requirement. The cathodes were heated at 120 °C for 10 h to remove moisture. Cells were assembled in argon filled glove box with cathode electrode, lithium metal disc as anode, 1 M LiPF_6_ solution in 1:1:1, v/v/v mixture of ethylene carbonate, dimethyl carbonate, and ethyl methyl carbonate (EC:DMC:EMC) and micro-porous polyethylene was used as separator. The cycling test was carried out at 25 °C in the voltage range of 3.0–4.3 V at various current densities by using electrochemical workstation (Won A-Tech, WBCS 3000 L).

Cyclic voltammetry (CV) was performed on VMP3, Bio-Logic electrochemical workstation by using three electrode system pouch cell in the range of 3.0–4.3 V at a scan rate of 0.1 mVs^−1^. The counter and reference electrode are of lithium metal attached on copper sheet were used along with the working electrode. Electrochemical impedance spectroscopy (EIS) test was performed by using as electrochemical workstation (Bio-Logic, VSP-300) in the frequency range of 10^−3^ – 10^+7^ Hz.

## Results and Discussion

**Figure 2:**
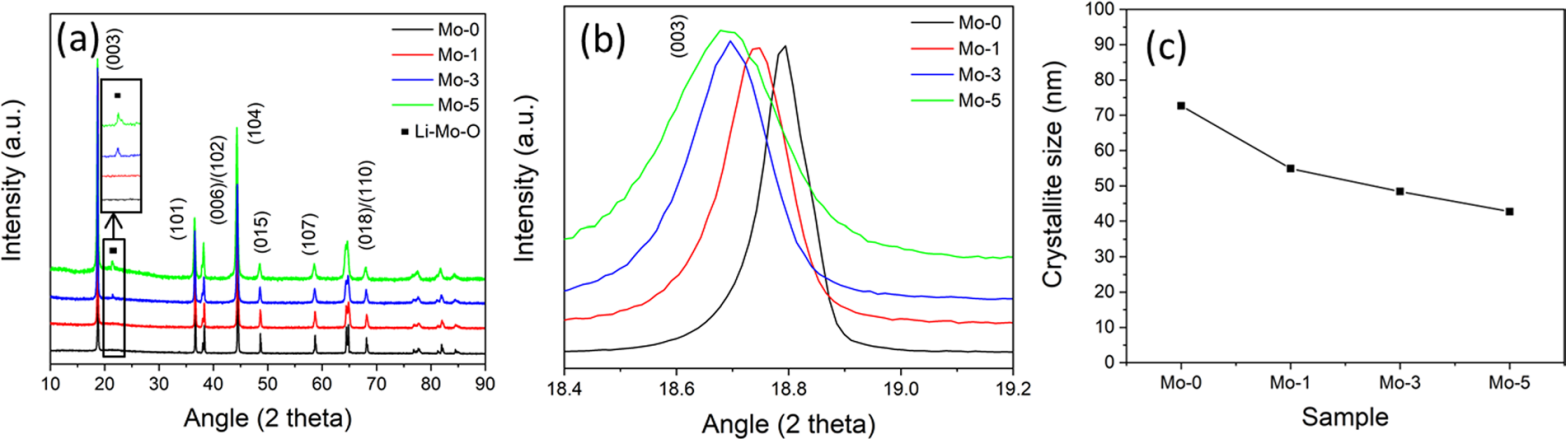
XRD diffraction patterns of (**a**) Mo-0 and Mo-modified samples, (**b**) (003) diffraction peak of samples, (**c**) crystallite size measurement of samples.

The XRD patterns of the samples are shown in Fig. 2(a). All the peaks are indexed to layered hexagonal α-NaFeO_2_ structure (R-3m space group) which is a clear indication that Mo is doped in the NCM. From the inset in Fig. 2(a), there is an impurity peak at around 21° in Mo-3 and Mo-5. By indexing, we conclude this peak belongs to the series of Li-Mo-O compounds^29^. The presence of Li-Mo-O suggests that excess amount of the dopant in Mo-3 and Mo-5 samples combines with the residual Li and oxygen over the secondary particle surface and then forms compound^31^. However, the amount of Li-Mo-O is not overmuch in Mo-1. Therefore, we could not find the evidence of Li-Mo-O in Mo-1. Figure 2(b) shows the magnified region of (003) peak, shifting of peaks to low angle for doped samples are an indication of lattice parameters expansion due to the Mo doping in the layered structure^22,29^. Therefore, the increased lattice spacing of synthesized samples enables the easier and faster Li ion diffusion, which enhances the electrochemical performances. Figure 2(c) shows the variation of crystallite size in different samples. It has been reported, the presence of secondary phase particles at the grain boundaries can hinder the grain growth^32,33^. Lin *et al*. has reported that Mo can refine the grain growth while pinning the grain boundaries^34^. It is clear that, Mo-doping suppresses the particle size growth that is why the crystallite size is also reduced by increasing the doping content in this study.

As shown in Supplementary Fig. S1 and Table 1, Rietveld refinement was conducted to study the changes in the lattice parameters with the increasing Mo content. As shown in Table 1, the lattice parameters of *a* and *c* increases with the increase in Mo samples which validates the Fig. 2(b) where the (003) peaks shifts to the lower angle. Moreover, with the increase in Mo amount the cell volume also grows as shown in Table 1. This can also ascribe to the larger ionic radii of Mo^6+^ (0.73 Å) than Mn^4+^ (0.53 Å) and Co^3+^ (0.545 Å)^28,29^. The increased lattice parameters help to enhance the rate capability and Li^+^ diffusion during cyclability^35,36^. As Mo^6+^ ionic radius (0.73 Å) is bigger than Mn^4+^ (0.53 Å) and Co^3+^ (0.545 Å) and slightly smaller than Li^+^ (0.76 Å), which is why there are good chances of Mo^6+^ settling inside lithium-slab and it can stabilize the structure for smooth Li ion diffusion during charge-discharge process^28,29^. The intensity ratio I_(003)_/I_(104)_ can be used to identify the cation disorder^37,38^. It’s a convention that cation mixing is reduced when the intensity ratio is above 1.2, indicates that the material has better degree of cation order^24,29,39^. Although, the ratios in our samples are above 1.2. After Mo modifying the synthesized samples shows hexagonal ordering, layered and crystallinity of the cathode material which can be identify from the splitting of (006)/(102) and (018)/(110) peaks^24,28,40^ as shown in supplementary Fig. S2(a),(b). To further verify the coating, we have conducted acid etching of the Mo-1 and Mo-5 sample by 0.1 M HCl solution. The XRD diffraction of acid etched Mo-1 and Mo-5 samples are shown in supplementary Fig. S3. The Li-Mo-O peak is absent after the acid treatment which inferred that during acid etching the coating compound is washed out from secondary particle surface. The (003) peak of acid etched Mo-1 and Mo-5 is still at lower angle as compared to Mo-0 (supplementary Fig. S3(b)) which confirms that Mo is still doped. Therefore, we can conclude that Mo is not only doped but as well coated on the NCM84.

**Table 1:** Lattice parameters of samples from XRD patterns.

Sample #	*a* (Å)	*c* (Å)	V (Å)^3^	I_003_/I_104_
Mo-0	2.8740	14.2014	101.6595	1.94
Mo-1	2.8750	14.2154	101.6868	1.92
Mo-3	2.8762	14.2180	101.8600	1.88
Mo-5	2.8810	14.2200	102.2200	1.65

**Figure 3:**
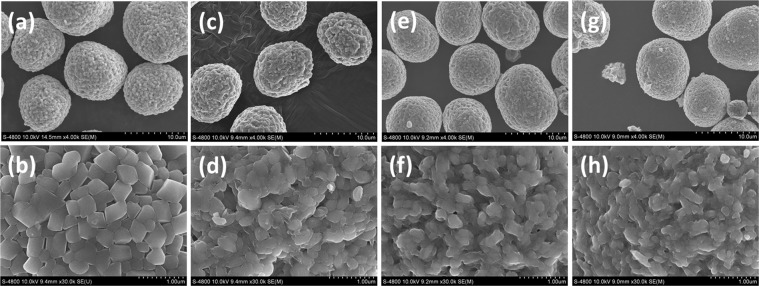
FESEM images of Mo-0 and Mo-modified NCM84 (**a,b**) Mo-0, (**c,d**) Mo-1, (**e,f**) Mo-3, (**g,h**) Mo-5 samples.

Figure 3(a)–(h) show the SEM images of as-prepared samples. All the samples consist on spherical shape secondary particle of around 9–10 μm which is further consist on primary particles. Furthermore, it is obvious to see the difference of primary particle size between Mo-0 and Mo-modified samples. The Mo-0 primary particle size is in the range of 400–500 nm while the modified samples are 200–300 nm. It can be inferred that the doping of Mo reduced the particle size which may be due to the slow grain growth of the doped samples^33^. This suppression of primary particle size is beneficial for cathode electrode because smaller particle size can increase the contact area between the cathode electrode and electrolyte. In addition, this can also decreases the lithium diffusion distances which can improve the de/intercalation for the lithium ion, resulting in excellent cyclability^13^. Internal stresses can grow-up during doping which can destroy the particles while the Mo-modified samples maintain their shape in the current work^29,41^. Moreover, it is interesting that as the amount of Mo is increased the surfaces (Fig. 3(d),(f),(h)) of primary particles are more like wetted and fused, because the Mo is too much to insert into the interior of crystal structure so the remaining Mo is also coated as well^29,31^. EDS mapping of Mo-1 was carried out to check the distribution of Mo in the sample. The supplementary Fig. S4(a) shows the SEM image of secondary particle used to examine the distribution of Mo, Ni, Co and Mn as presented in supplementary Fig. S4(b–e). As shown in supplementary Fig. S4(b), Mo is homogeneously distributed throughout the secondary particle surface indicating that Mo is effectively doped in and Li-Mo-O coated on the particle surface. The SEM images of the acid etched Mo-1 sample is shown in supplementary Fig. S5(e),(f). The acid reacts with the coating and etched the secondary particles as a result the clear edges of primary particles are observed in supplementary Fig. S5(e),(f). This is the clear confirmation of the coating along with doping. For the further validation, EDS mapping of acid-etched Mo-1 sample was measured. The supplementary Fig. S6(a) shows the SEM image of acid etched Mo-1 sample while supplementary Fig. S6(b–e) shows the distribution of Mo, Ni, Co and Mn. Even after the etching, the remaining Mo is only doped one which has been found uniformly distributed in elemental mapping results. Therefore, we can conclude that Mo is doped as well as coated in this study.

**Figure 4:**
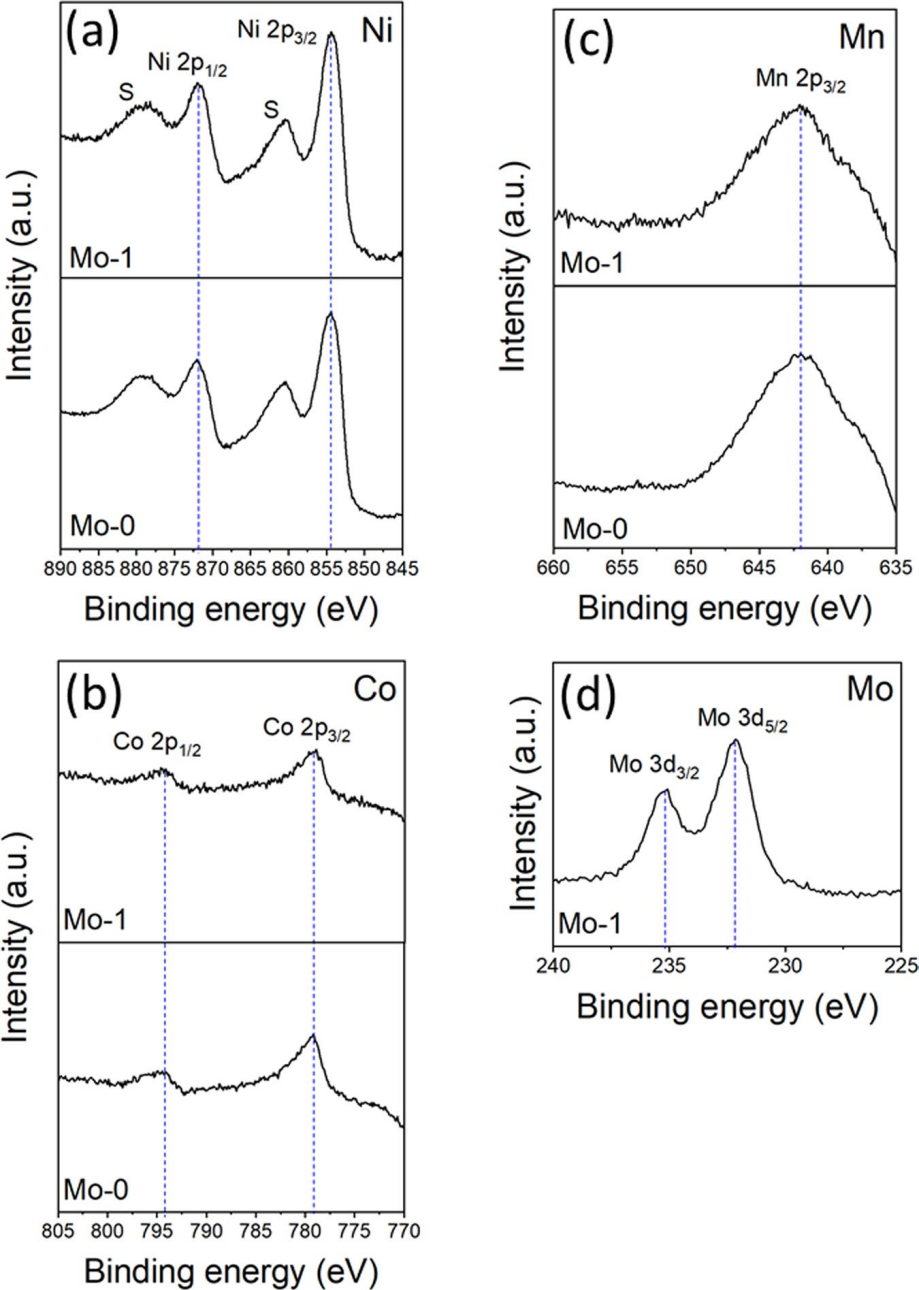
XPS spectra of (**a**) Ni 2p, (**b**) Co 2p, (**c**) Mn 2p and (**d**) Mo 3d for Mo-0 and Mo-1 sample.

X-ray photoelectron spectroscopy (XPS) was used to further study the oxidation states of the metal elements (Ni, Co, Mn and Mo) in the synthesized samples. The XPS spectra of Mo-0 and Mo-1 are shown in Fig. 4. As compared to the Mo-0, the binding energies of Ni_2p_, Co_2p_ and Mn_2p_ have no significant changes even after the Mo-modification. Figure 4(a) shows the obvious peaks at 854.64 eV and 871.74 eV are assigned to the Ni 2p_3/2_ and Ni 2p_1/2_^42^. As shown in Fig. 4(b,c) the binding energy peaks of Co 2p_3/2_ and Mn 2p_3/2_ appears at 779.04 eV and 642.08 eV, which confirms the oxidation state of +3 and +4 for the Co and Mn, respectively^28^. Due to small amount of Mo, the corresponding peak of Mo was not found in the XRD (Fig. 2). However, the spectrum of Mo_3d_ confirms its presence as shown in Fig. 4(d). The binding energy peaks of Mo 3d_5/2_ and Mo 3d_3/2_ are found at 232.18 eV and 235.38 eV, respectively, which implies the valence state of +6^35^. This inferred that Mo-modified samples have a coating of Mo-compound (i.e. Li-Mo-O) because the XPS is an elemental surface characterization technique^29^. From XPS results, we can conclude that prepared samples are Mo-modified samples.

**Figure 5:**
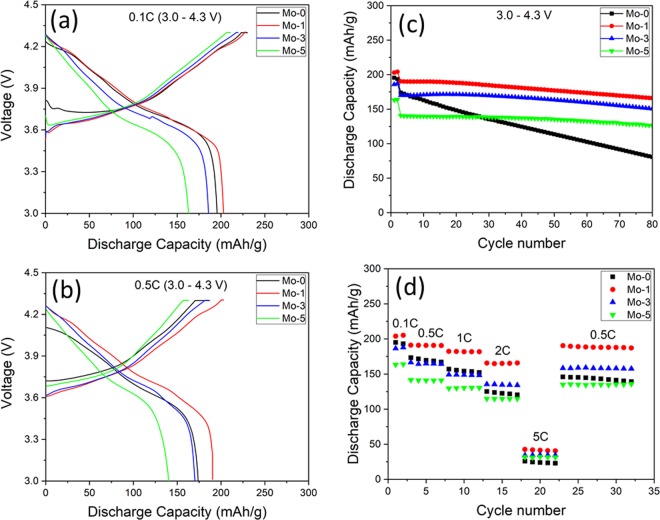
Initial charge-discharge curves (**a**) 0.1 C and (**b**) 0.5 C rate, (**c**) cycling performance, (**d**) Rate performance of Mo-0, Mo-modified samples between 3.0–4.3 V.

To investigate the effect of Mo-doping on electrochemical properties, initial charge-discharge curves of all samples at 0.1 C and 0.5 C rate between 3.0–4.3 V at 25 °C has been carried out, as shown in Fig. 5(a),(b). The specific discharge capacities for Mo-0, Mo-1, Mo-3 and Mo-5 at 0.1 C are 195 mAh.g^−1^, 205 mAh.g^−1^, 185 mAh.g^−1^,163 mAh.g^−1^, while at 0.5 C are 173 mAh.g^−1^, 191 mAh.g^−1^, 170 mAh.g^−1^, 140 mAh.g^−1^, respectively. It is clear from the Fig. 5(a),(b), polarization decreases after certain amount of Mo-modifying the NCM material but later on the excess Mo increase the polarization like in the case of Mo-3 and Mo-5^29^. Among all the samples, Mo-1 shows the highest specific capacity due to the higher material utilization and fast electrode kinetics^43^. However, Mo-3 and Mo-5 lower discharge capacity is due to the high amount of dopant and increase in coating thickness^15^. As the Mo-dopant is increased, Li-intercalation into the surface-slab leads to the capacitor behavior which increases as the crystallite size decreases^44^. Both the enlargement in surface area and reduction in crystal lattice enhanced the capacitor behavior, that results in decreased specific capacity due to the reduction in internal sites^45^.

The cycling performance of all the samples at 0.1 C (2 cycles) and 0.5 C (78 cycles) rate at a cut-off voltage of 3.0–4.3 V at 25 °C are presented in Fig. 5(c). The specific discharge capacity at 80^th^ cycle and capacity retention of all the samples are; Mo-0 is 81 mAh. g^−1^ (46.5%), Mo-1 is 165 mAh. g^−1^ (87.2%), Mo-3 is 150 mAh. g^−1^ (88.5%), Mo-5 is 126 mAh. g^−1^ (89.6%). However, during cycling Mo-modified samples show quite promising results while the Mo-0 shows poor capacity retention from the beginning. The low specific discharge capacity in case of Mo-0 is due to deteriorated structure and large primary particles (in conjunction with SEM image) which result in poor structural stability and slow kinetics for the lithium ion de/intercalation. In case of modified samples, Mo refined the primary particle size and the structure morphology sustained its spherical morphology. Moreover, the presence of Mo^6+^ in the lithium slab and Li-Mo-O on the surface provides the structural stability during delithiation which leads to good cyclability^28,35^. Also, the bond length of Mo-O (2.055 Å) is large as compared to Ni-O (1.975 Å), Co-O (1.921 Å) and Mn-O (1.947 Å)^28,46^ which enlarges the lithium slab spacing that can enhance the conduction of Li^+^ during cycling. All these combine to be responsible for the modified samples to exhibit stable cycling performance as compared to Mo-0. However, slightly high amount of doping blocks the lithium ion passageway that is responsible to decrease the discharge capacity of Mo-3 and Mo-5^15^. In addition, the coating may segregate the active material and electrolyte which would reduce the chances of dissolution of transition metals that can suppresses the irreversible capacity loss^29^. This brings the enhancement of cyclability as found in Mo-3 and Mo-5 but at the cost of low discharge capacity as compared to other samples. The Mo-1 shows better results in terms of high discharge capacity and cyclability. Another important factor of the LIB for energy storage applications is the rate capability. Figure 5(d) exhibits the rate capability of all the samples from 0.5 C to 5 C in the voltage range of 3.0–4.3 V. The samples are cycled 5 times at each rate. The retention difference of the samples is increasing as the C-rate is increases. Mo-0 shows better results at low rate as compared to Mo-3 and Mo-5 but at higher rates (5 C) the discharge capacity drops down to lowest. The major factor is the sluggish kinetics of the lithium ion diffusion that originated from the high internal stress. Mo-1 performs well at all C-rates and the discharge capacities are 205 mAh.g^−1^, 191 mAh.g^−1^, 182 mAh.g^−1^, 165 mAh.g^−1^ and 42 mAh.g^−1^ at 0.1, 0.5, 1, 2 and 5 C respectively. Furthermore, only Mo-1 recovers the same initial specific capacity when the C-rate is brought back to 0.5 C. It can be inferred that the optimized amount of doping and coating can enhance the rate capability significantly, which is consistent with the previous study by zhang *et al*.^29^.

**Figure 6:**
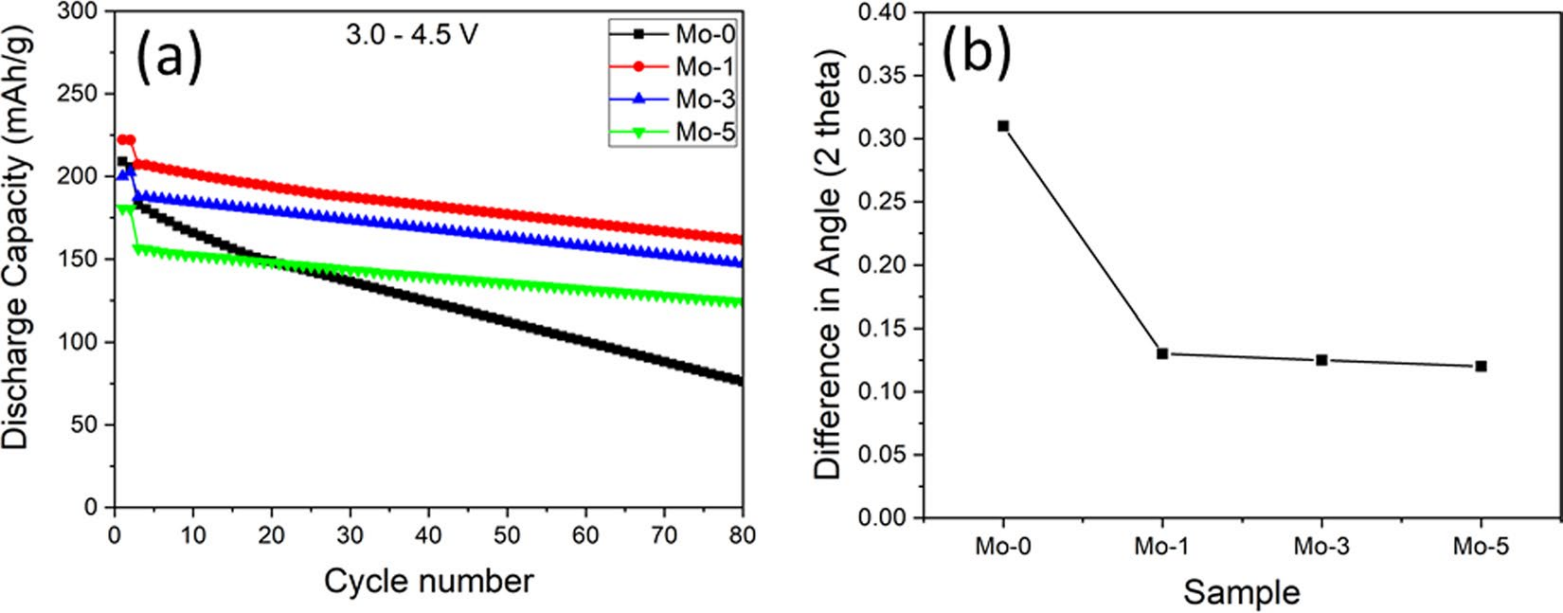
Cycling performance of Mo-0 and Mo-modified samples between 3.0–4.5 V, (**b**) Difference in (003) diffraction peak of 2θ between fresh and cycled electrodes (3.0–4.5 V).

**Figure 7:**
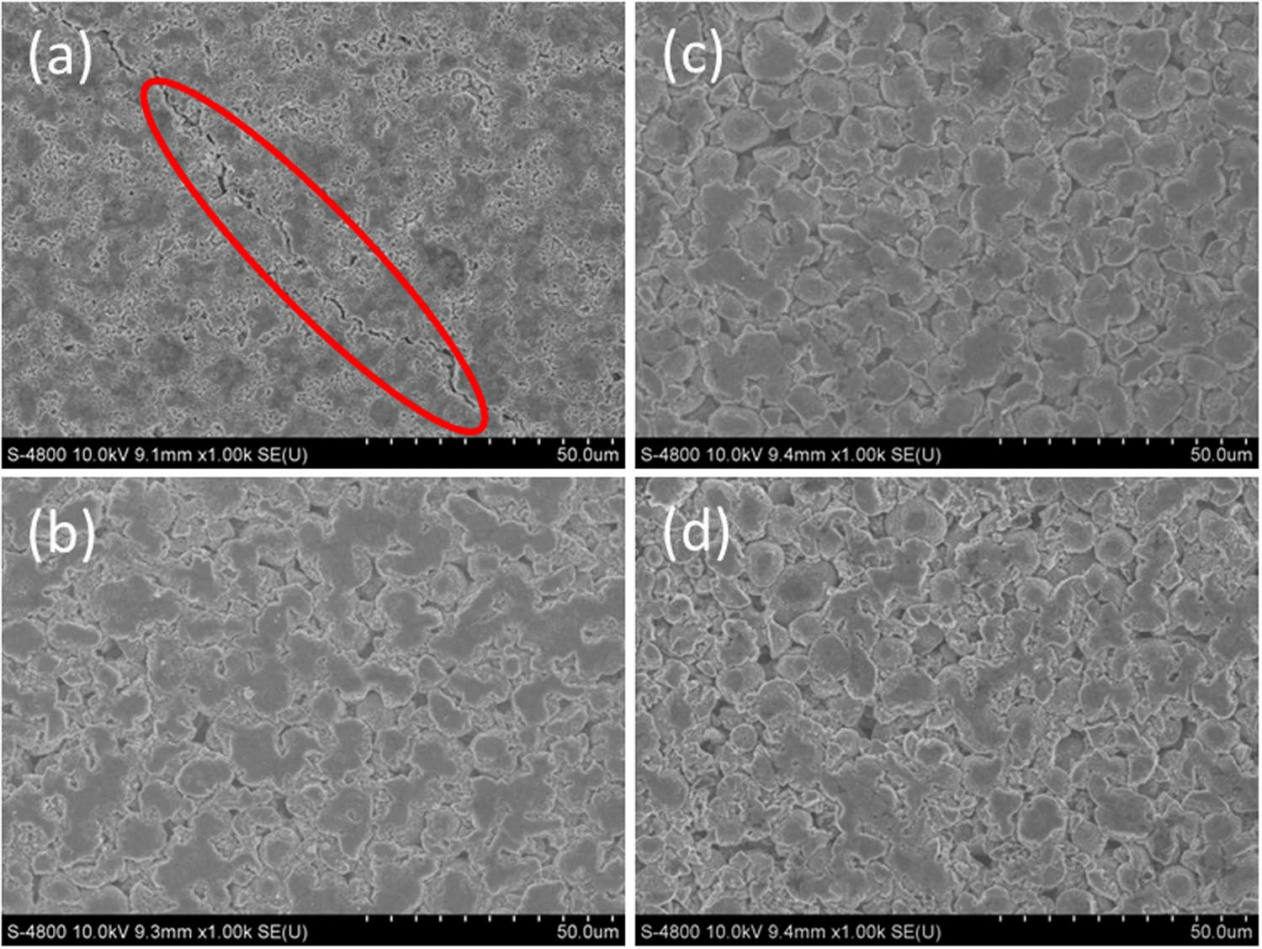
FESEM images of cycled samples between 3.0–4.5 V (**a**) Mo-0, (**b**) Mo-1, (**c**) Mo-3 and (**d**) Mo-5.

Figure 6(a) shows the cyclability of the samples at cut-off voltage between 3.0–4.5 V, at 25 °C for 80 cycles. Even at higher cut-off voltage the capacity retention of Mo-modified samples is increase as compared to the Mo-0. The capacity retention of Mo-0, Mo-1, Mo-3 and Mo-5 are 41.6%, 77.8%, 78.4% and 79.3%, respectively. It is evident that when the voltage range becomes wider the cyclic performance of the electrodes decreases^47^. Mo-0 (1^st^ cycle 209 mAh.g^−1^) shows low discharge capacity and poor cycling behavior as compared to the rest because, (1) structure goes through severe distortion during highly charged state, and (2) the longer channels for lithium ion diffusion. Mo-1, Mo-3 and Mo-5 capacity retention is quite close but still the discharge capacity of Mo-1 (222 mAh.g^−1^) is higher than the Mo-3 (200 mAh.g^−1^) and Mo-5 (180 mAh.g^−1^). The increase in the coating thickness, can be estimated from inset of Fig. 2 (a) is the main reason behind improved cyclability because it acts like an protective layer between the particles and electrolyte which suppresses the side reactions^29^. Moreover, optimized amount of doping can keep the channels open for fast and smooth lithium-ion diffusion while thin coating layer is the main reason behind high discharge capacity and improved cyclic performance of Mo-1. The supplementary Fig. S7 shows the comparison of XRD patterns of fresh and cycled samples in order to investigate the effect of high voltage (3.0–4.5 V) cycling on the host structure. We can confirm that the shifting of (003) diffraction peak towards low angle during cycling (3.0–4.5 V); the data is summarized in Fig. 6(b). The peak shifting is more than twice in case of Mo-0 as compared to modified samples. This can be explained on the basis of two facts; first is the cracking of particles (Fig. 7) and second is the continuous expansion of crystal lattice which causes the breakdown of host structure^48,49^. Figure 7 shows the SEM images of samples cycled between 3.0–4.5 V. From the images, a crack is visible (encircled in red) on the surface of Mo-0. However, in case of Mo-modified samples the surface is smooth, without cracks and they sustain their structural integrity owing to buffer space which results in the enhanced cycling performance. The reason behind cracking in Mo-0 is its primary particle growth; when densely packed particles goes through anisotropic lattice contraction and expansion during charge-discharge process that exerts huge strain on the particles and grain boundaries; as a result crack initiates and propagate^50,51^. This crack can greatly affect the battery performance in the long term, i) phase transformation and side reaction can lead to the creation of new surface which reacts with electrolyte, ii) loss of grain to grain bonding; electrical conductivity can decrease and also loss of active material due to particle pulverization^52^. As a result, the electrode resistance will increase and capacity will decrease quickly. That’s why, cracks are considered as notorious in the battery performance^53^.

**Figure 8:**
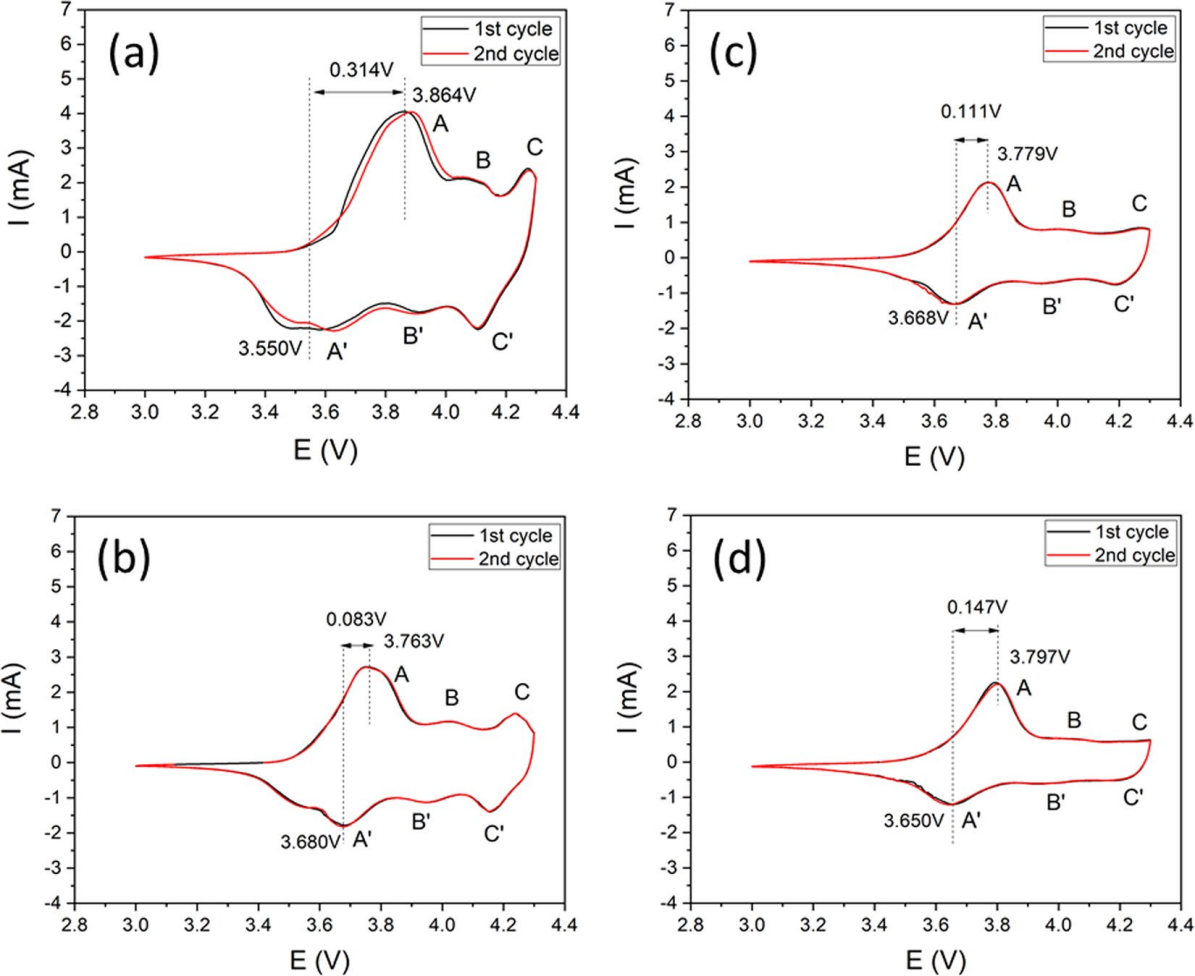
CV profiles of Mo-0 and Mo-modified samples (**a**) Mo-0, (**b**) Mo-1, (**c**) Mo-3 and (**d**) Mo-5.

Ni-rich cathode materials are structurally unstable due to release of oxygen atom that mutates the structure due to oxygen defects which are caused because of the low bonding energy of Ni^3+^ and O^2-^^54^. For further understanding the effect of Mo-doping on the structural transformation, CV was performed in the range of 3.0–4.3 V at a scan rate of 0.1 mVs^−1^. Figure 8 exhibits the cyclic voltammograms of all the samples indicating that the addition of Mo has successfully suppress the phase transformation in modified samples. There are mainly three pairs of peaks which are an indication of phase transformation, i.e. A-A’ (hexagonal to monoclinic; H1 to M), B-B’ (Monoclinic to hexagonal; M to H2) and C-C’ (hexagonal to hexagonal; H2 to H3) during lithium de/intercalation^22^. No transition from hexagonal to monoclinic is observed as represented by A-A’; which is related to the oxidation of Ni^2+/3+^ to Ni^4+^^13^. Addition of Mo greatly reduces the intensities of B-B’ and C’-C’. As reported in literature, the volume changes in Ni-rich cathode is due to transition between H2 to H3 which causes the pulverization in secondary particles resulting in capacity drops^22^. It is clear, that by increasing the Mo the transformation peaks suppressed which concludes that detrimental phase transformations have been reduced^36^. Mo-5 shows superior electrochemical stability as compared to all of the samples at the expense of discharge capacity because the dopant amount is high as well as coating is very thick which has isolates the active material and electrolyte. The voltage difference between oxidation and reduction peak for the first cycle of all the samples are 0.314 V (Mo-0), 0.083 V (Mo-1), 0.111 V (Mo-3) and 0.147 V (Mo-5).

**Figure 9:**
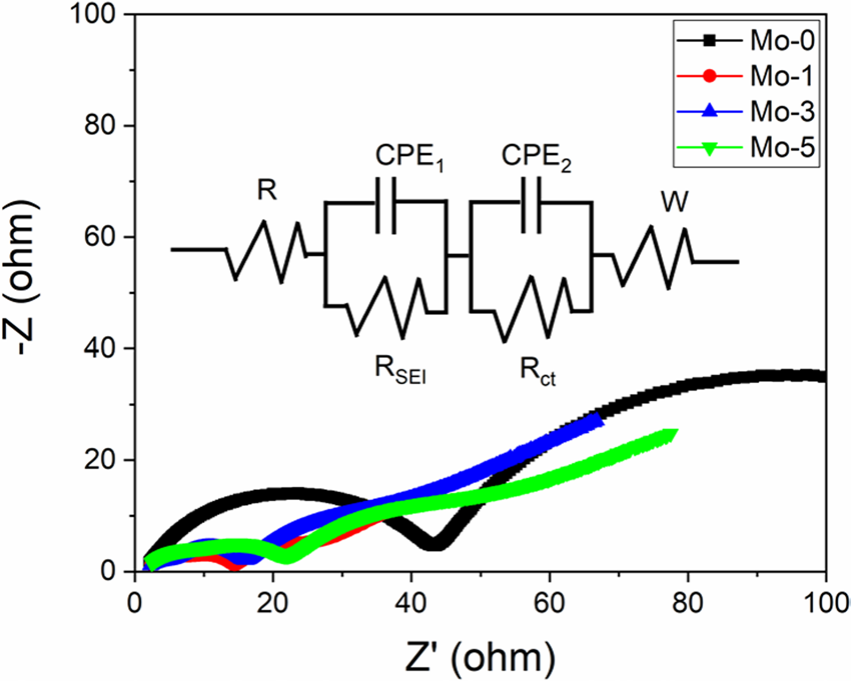
Nyquist plots of Mo-0, Mo-1, Mo-3 and Mo-5 samples.

Figure 9 shows the Nyquist plots of the samples after 10 cycles. The Nyquist plots are consist of three regions; a straight line at low frequency, one at the intermediate and two semicircles at higher frequency. The ohmic resistance (R_s_) is the intercept of EIS curves on real axis. Semicircle at the intermediate frequency shows the charge transfer resistance (R_ct_) which is an important data to explain the electrochemical performance because the R_ct_ is mainly contributes in the electrochemical reactions. Two semicircles at the high frequency represent the lithium ion diffusion resistance (R_SET_) and the electronic interface resistance (R_e_)^29^. The R_ct_ of Mo-0 is 43.3 Ω while for Mo-1, Mo-3 and Mo-5 is14.3, 16.1 and 22.0Ω after 10th cycle. Since the higher R_ct_ value indicates the kinetic barrier for the lithium ion diffusion which enhances the capacity fading during cycling^3,28,55^. Mo-1 shows the least R_ct_ value which is one third of the Mo-0 because Mo-modifying can suppress the increase in R_ct_, enabling the fast and smooth lithium ion diffusion. Therefore, we can conclude that Mo-doping is beneficial to enhance the structural stability of NCM84 material.

## Conclusions

Ni-rich cathode material LiNi_0.84_Co_0.11_Mn_0.05_Mo_x_O_2_ (x = 0, 1, 3, and 5 wt. %) has been prepared by solid-state method. The Mo-modified samples show spherical shape secondary particle while the primary particle size reduced to a large extent as compared to the Mo-0 sample. The reduction in primary particle size makes the short lithium diffusion passageway leading to superior cyclability and rate capability. The presence of Mo in lithium slab and coating on the particle surface provides structural stability. Mo-1 and Mo-3 showed relatively similar capacity retention but the discharge capacity is quite high in Mo-1. Among all samples, the Mo-1 show the best electrochemical performances originated from optimized amount of dopant in lithium slab and coating. Therefore, the Mo-1 can be regarded as a promising candidate for high-energy LIB cathode.

## Supplementary information

Supplementary Information.
