# Supplementary material for: Influence of Mo addition on the structural and electrochemical performance of Ni-rich cathode material for lithium-ion batteries
Source: Sci Rep. 2020 May 22;10:8562. doi: 10.1038/s41598-020-64546-8 (PMC7244510; doi:10.1038/s41598-020-64546-8)
Supplement: Supplementary file 1 — Supplementary Information. [file 41598_2020_64546_MOESM1_ESM.docx]

**Supplementary Information**

**Influence of Mo addition on the structural and electrochemical performance of Ni-rich cathode material for lithium-ion batteries**

Tahir Sattar^a,b,c,1^, Seung-Hwan Lee^a,1^, Bong-Soo Jin^a^, Hyun-Soo Kim ^a,*^

^a^Next Generation Battery Research Center, Korea Electrotechnology Research Institute (KERI), Changwon, Republic of Korea.

^b^University of Science and Technology, Daejeon, Republic of Korea.

^c^Faculty of Materials and Chemical Engineering, Ghulam Ishaq Khan Institute of Engineering Sciences and Technology, Topi, Khyber Pakhtunkhwa, Pakistan.

**^*^Corresponding author’s email:** [hskim@keri.re.kr](mailto:hskim@keri.re.kr%20) **Tel: +82-55-280-1699 Fax: +82-55-280-1590**

**
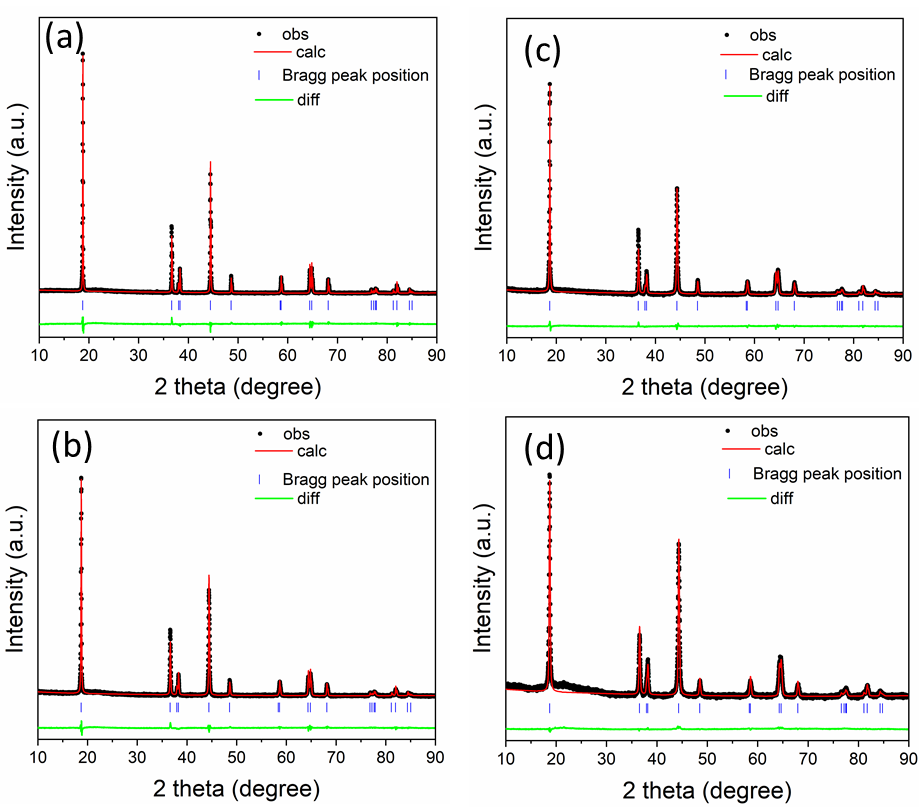
**

Figure S1 Rietveld refinement of (a) Mo-0, (b) Mo-1, (c) Mo-3 and (d) Mo-5.


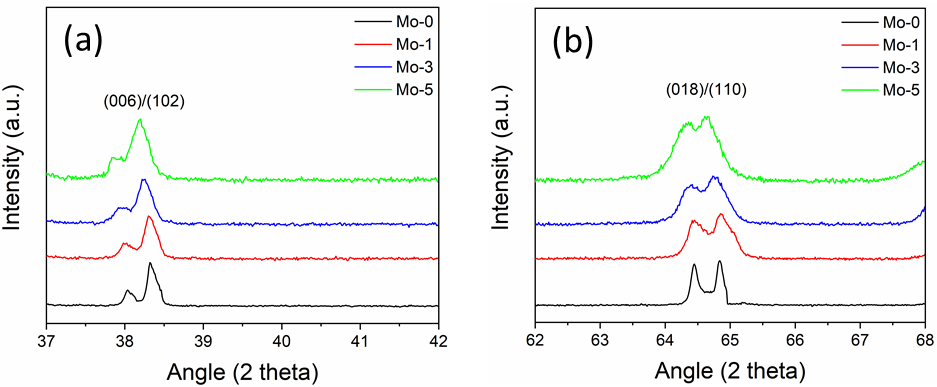


Figure S2 XRD diffraction pattern showing splitting of (a) (006)/(102) and (b) (018)/(110) peaks of Mo-0, Mo-modified samples.


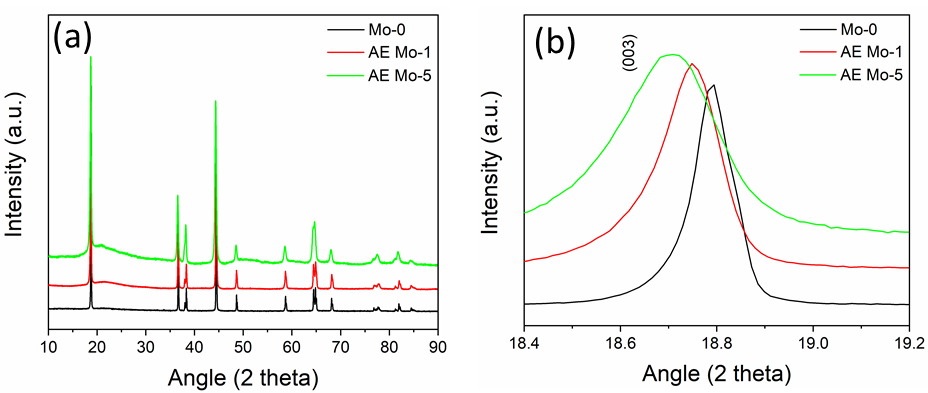


Figure S3 XRD diffraction patterns of (a) Mo-0, acid-etched Mo-1 and acid-etched Mo-5, (b) (003) diffraction peak of Mo-0, acid-etched Mo-1 and acid-etched Mo-5 samples.


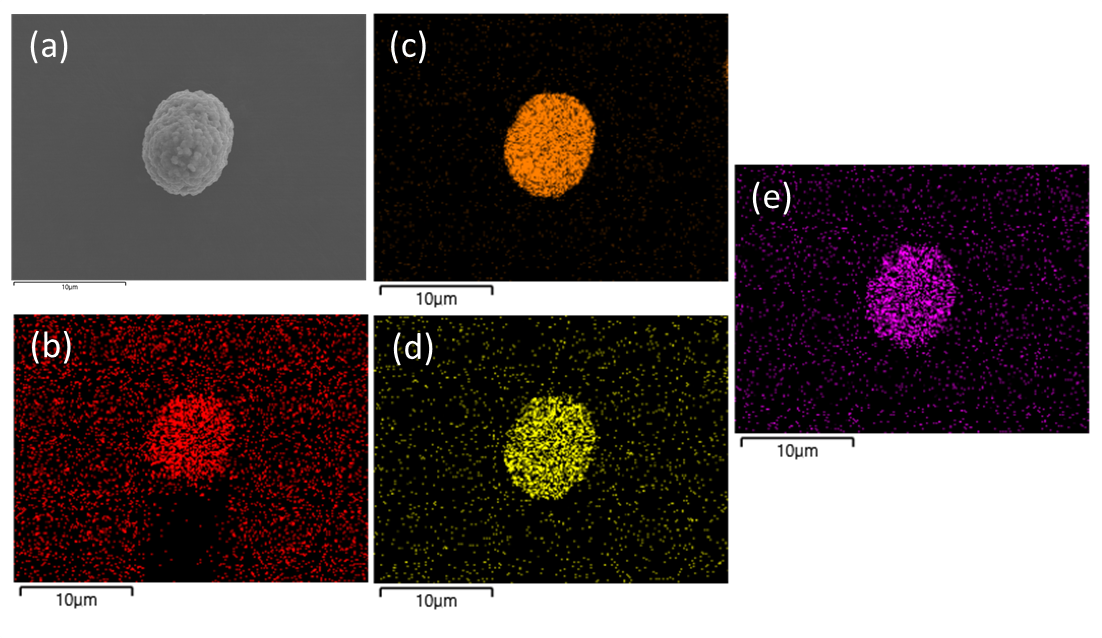


Figure S4 (a) SEM image of Mo-1 and EDS mapping showing distribution of elements (b) Mo, (c) Ni, (d) Co and (e) Mn


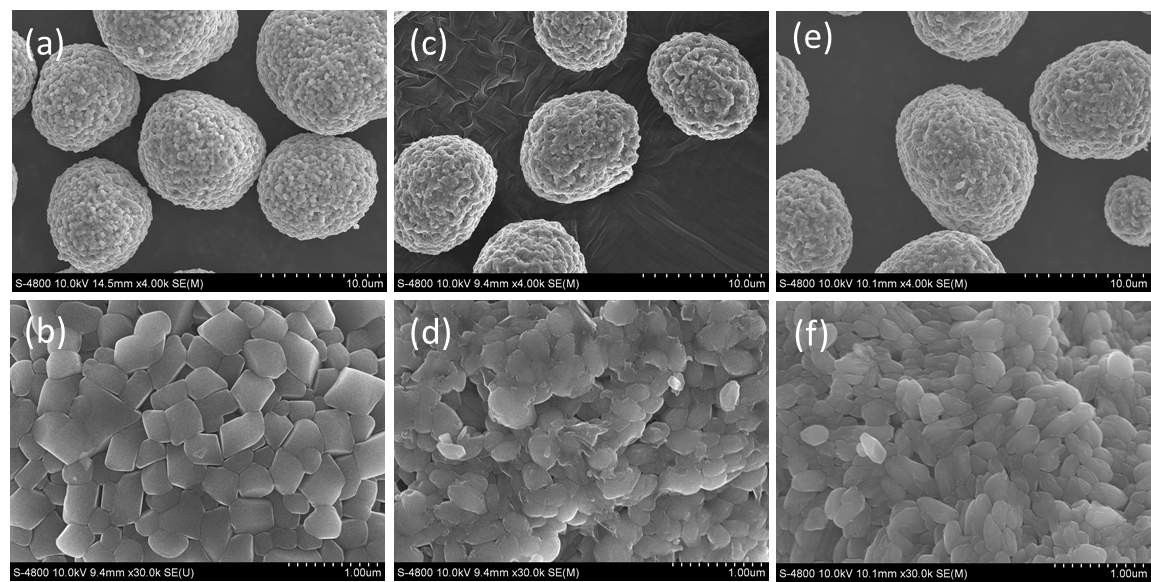


Figure S5 SEM images of (a,b) Mo-0, (c,d) Mo-1 and (e,f) acid-etched Mo-1.


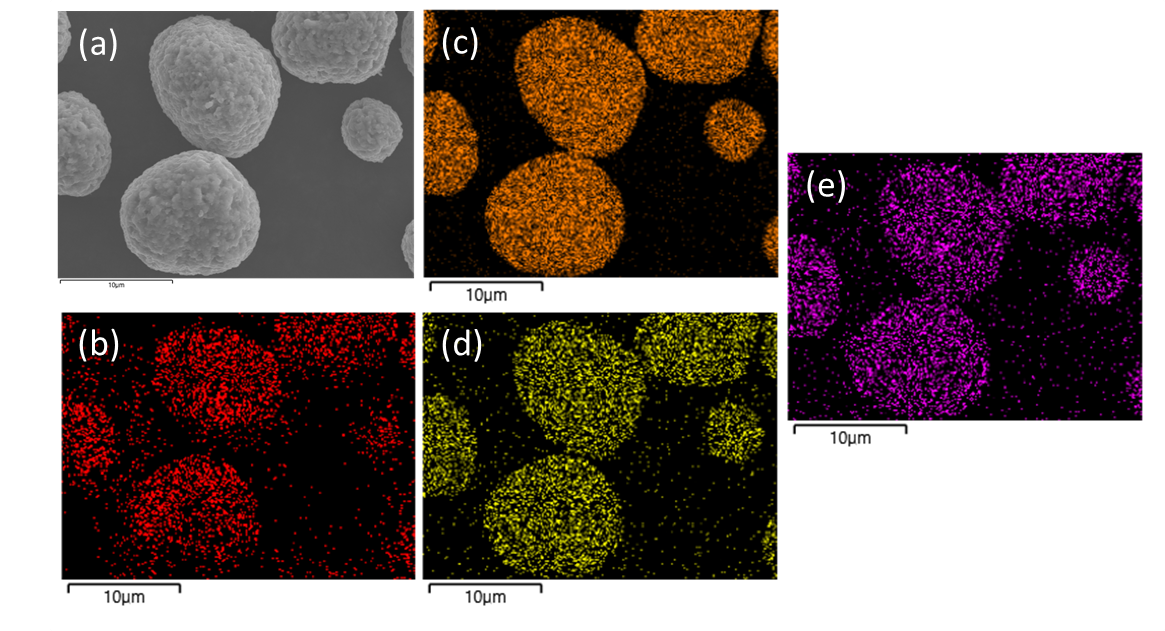


Figure S6 (a) SEM image of acid etched Mo-1 sample and EDS mapping showing elemental distribution (b) Mo, (c) Ni, (d) Co and (e) Mn.


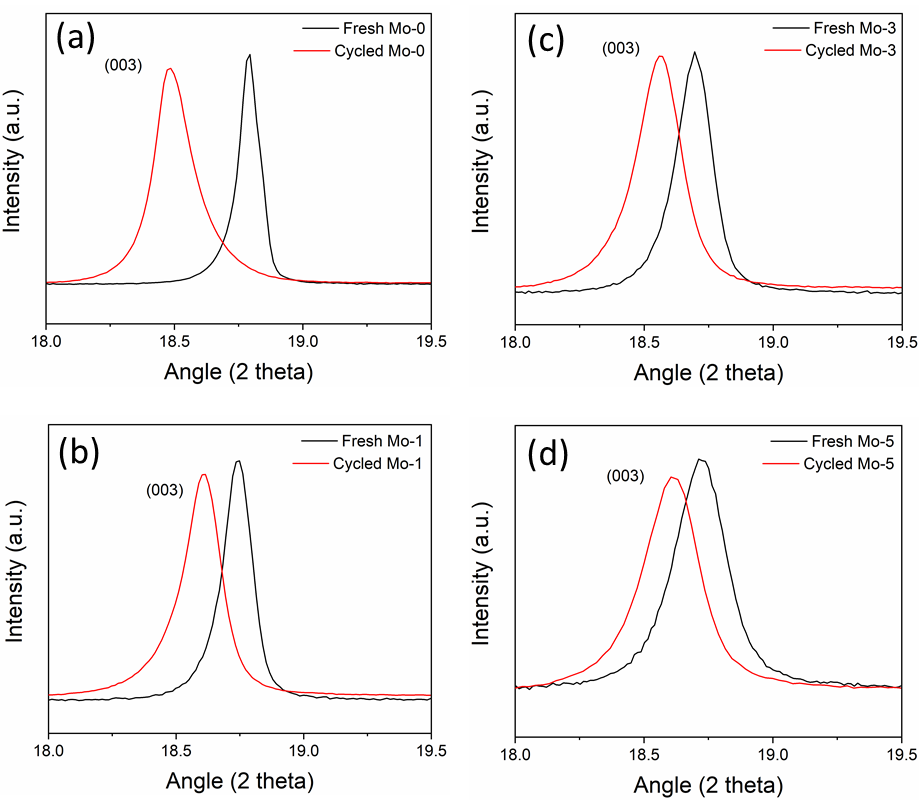


Figure S7 XRD diffraction pattern showing shifting of (003) peaks before and after 4.5V cycling.
